# Orbital Angular Momentum (OAM) Antennas via Mode Combining and Canceling in Near-field

**DOI:** 10.1038/s41598-017-13125-5

**Published:** 2017-10-09

**Authors:** Woo Jin Byun, Hyung Do Choi, Yong Heui Cho

**Affiliations:** 1 Radio Resource Research Group, Electronics and Telecommunications Research Institute (ETRI), Daejeon, 34129 Korea; 2 Radio Environment & Monitoring Research Group, Electronics and Telecommunications Research Institute (ETRI), Daejeon, 34129 Korea; 3 School of Information and Communication Engineering, Mokwon University, Daejeon, 35349 Korea

## Abstract

Orbital angular momentum (OAM) mode combining and canceling in the near-field was investigated using a Cassegrain dual-reflectarray antenna composed of multiple microstrip patches on the main and sub-reflectarrays. Microstrip patches on dielectric substrates were designed to radiate the particular OAM modes for arithmetic mode combining, where two OAM wave-generating reflectarrays are very closely placed in the near-field. We conducted near-field antenna measurements at 18 [GHz] by manually replacing the sub-reflectarray substrates with different OAM mode numbers of 0, ±1, when the OAM mode number of the main reflectarray was fixed to +1. We subsequently checked the azimuthal phase distributions of the reflected total electromagnetic waves in the near-field, and verified that the OAM waves mutually reflected from the main and sub-reflectarrays are added or subtracted to each other according to their OAM mode numbers. Based on our proposal, an OAM mode-canceling reflectarray antenna was designed, and the following measurements indicate that the antenna has a better reflection bandwidth and antenna gain than a conventional reflectarray antenna. The concept of OAM mode canceling in the near-field can contribute widely to a new type of low-profile, broad-reflection bandwidth, and high-gain antenna.

## Introduction

It is well known that a light beam can carry additional orbital angular momentum (OAM) and its optical characteristics have been demonstrated based on Laguerre-Gaussian laser modes^1^. In addition, the various physical aspects and applications of OAM modes have been introduced^2^. OAM-based communication systems, originally studied in the field of optics, have been extended to the microwave bands^3–5^. Based on the polarization diversity and OAM mode multiplexing/demultiplexing, high-capacity OAM radios^6^ with $l=\pm 1,\pm 3$ were proposed and experimentally implemented at 28 [GHz], where *l* is the OAM mode number, and the achieved transmission distance of the proposed OAM link is 2.5 [m]. Utilization of multiplexed OAM modes is further extended to a traveling-wave slot antenna with ring cavity resonators^7^, and a Cassegrain reflector antenna with an OAM mode mux^8^ for use at 61 and 18 [GHz], respectively. Several studies were further conducted to discriminate an OAM radio from a multiple-input-multiple-output (MIMO) system^9,10^. When array elements are arranged to constitute a uniform circular array (UCA), an OAM radio composed of a UCA shows the same spectral efficiency as a conventional MIMO system below the Rayleigh distance^9^. However, by utilizing the space diversity of OAM modes, an OAM-based MIMO system theoretically increases the capacity gain more than a conventional MIMO system^10^. Most researches related to OAM communication systems have focused on far-field communication links^5–11^ with the same frequency and polarization to increase the channel capacity within limited communication resources. The near-field characteristics of particular OAM modes have recently been investigated^12–14^. For near-field radio communication with an enhanced capacity gain^9^, transmitting (Tx) and receiving (Rx) helicoidal reflectors ($l=0,\pm 1$) were set to face each other, and the OAM mode isolation between the Tx and Rx OAM mode numbers was experimentally proved^14^.

In this paper, we present the original idea that arbitrary OAM modes are arithmetically combined or canceled in both the near- and far-fields. To verify the OAM mode combining in the near-field, we designed and measured a Cassegrain dual-reflectarray antenna with closely placed main and sub-reflectarrays at 18 [GHz], which directly reflect and mix the predetermined OAM mode numbers, ${l}_{{\rm{main}}}=+1$ and ${l}_{{\rm{sub}}}=0,\pm 1$. Simulated and measured near-field distributions of the Cassegrain dual-reflectarray antennas show that the combined total phase patterns yield ${l}_{{\rm{tot}}}=0,+1,+2$ according to ${l}_{{\rm{sub}}}=+1,0,-1$ with the fixed ${l}_{{\rm{main}}}=+1$, respectively, and thus the OAM mode combining phenomenon occurring in the near-field is evident. In addition, a new low-profile OAM mode-canceling reflectarray antenna with ${l}_{{\rm{main}}}={l}_{{\rm{sub}}}=+1$ shows excellent broadband reflection and high antenna gain compared with a conventional reflectarray antenna.

## Results

### Combined OAM Modes in Near-field

**Figure 1:**
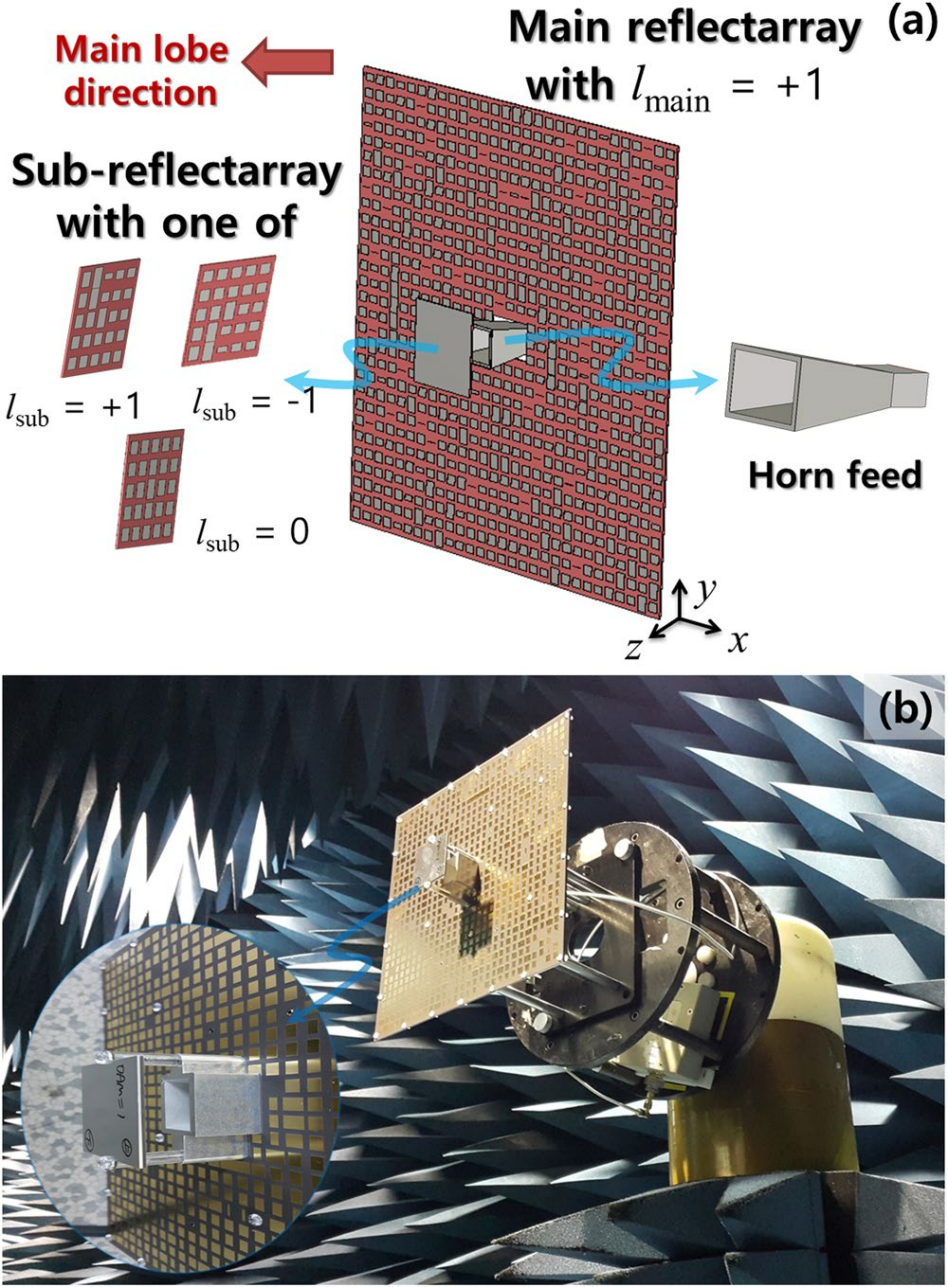
**(a)** Conceptual setup of a Cassegrain dual-reflectarray antenna with main and sub-reflectarrays to verify the mode combining and canceling in the near-field by manually replacing the sub-reflectarray substrates (${l}_{{\rm{sub}}}=0,\,\pm 1$). The main reflectarray substrate generated the fixed OAM mode number *l*_main_ = +1 for all simulations and measurements. **(b)** Image of far-field measurement setup with attached fabricated main and sub-reflectarrays. The reflectarray antennas were implemented using microstrip patches and fed by ordinary horn antennas.

A dual-reflector configuration^15,16^ is a good candidate for investigating the near-field characteristics of OAM modes owing to the fact that the usual distance between the main reflector and subreflector is quite small. We choose a Cassegrain dual-reflectarray antenna with the main and sub-reflectarrays composed of multiple microstrip patches on thin dielectric substrates, as illustrated in Fig. 1a. Reflectarray metasurfaces^17^ with microstrip patches were applied for the simultaneous generation of two OAM beams ($l=+1,+2$)^18^. Utilizing the standardized design procedures for an axially symmetric Cassegrain dual-reflector antenna^16^, the required reflectarray parameters for ordinary electromagnetic (EM) waves (*l* = 0) can be completely set. Next, we determined the reflected phase of each array element on the main and sub-reflectarrays for the generation of the desired OAM mode number. Based on the geometric phase equivalence [see the Supplementary Derivation of Equations (1) and (2)], a phase-matching condition, ${\psi }_{pq}$, for the $(p,q)$ th patch element on the main or sub-reflectarray shown in Fig. 1 was formulated as follows:

1 $${\psi }_{pq}={\rm{\Delta }}{\varphi }_{R}-{\rm{\Delta }}{\varphi }_{f}+l{\varphi }_{pq}={\psi }_{0}({\rho }_{pq})+l{\varphi }_{pq},$$

where ${\rm{\Delta }}{\varphi }_{f}$ and ${\rm{\Delta }}{\varphi }_{R}$ denote the phase shift of the direct and reflected rays caused by a feed and general reflector, respectively, *l* is the OAM mode number, ${\psi }_{0}(\rho )$ is a non-vortex phase term for ordinary EM waves (*l* = 0), and ${\varphi }_{pq}$ $[={\tan }^{-1}({y}_{pq}/{x}_{pq})]$ is the azimuthal position of the patch element at $({x}_{pq},{y}_{pq})$. The microstrip patch dimensions were determined in sequence based on the mapping relation^17^ between the patch length and ${\varphi }_{pq}$ (see Fig. 3 in the Supplement). The radiation behaviors of the dual-reflectarray antenna, shown in Fig. 1a, were measured in the near- and far-field ranges. Figure 1b illustrates the far-field measurement setup used to obtain the radiation patterns and antenna gains (see the Supplementary Measurement of Cassegrain Dual-reflectarray Antennas for the near-field measurement setup). Ordinary EM waves (*l* = 0) radiated from the horn feed, which is shown in the magnified inset of Fig. 1b, are reflected using a sub-reflectarray designed for a fixed OAM mode (${l}_{{\rm{sub}}}=0,\pm 1$). The effective deformation of a subreflector was proposed to generate the specific OAM mode^19^ ($l=+1$). The main reflectarray with ${l}_{{\rm{main}}}=+1$ OAM mode, which was placed very close to the sub-reflectarray, was used to apply the OAM mode combining of *l*_sub_ and *l*_main_. During the antenna experiments shown in Fig. 1b, the sub-reflectarray was manually replaced by a substrate reflecting one of the ${l}_{{\rm{sub}}}=0,\pm 1$ OAM modes for verification of OAM mode combining in the near-field. This indicates that the Cassegrain configuration allows the spatial mode combining to be constructed in the near-field.

**Figure 2:**
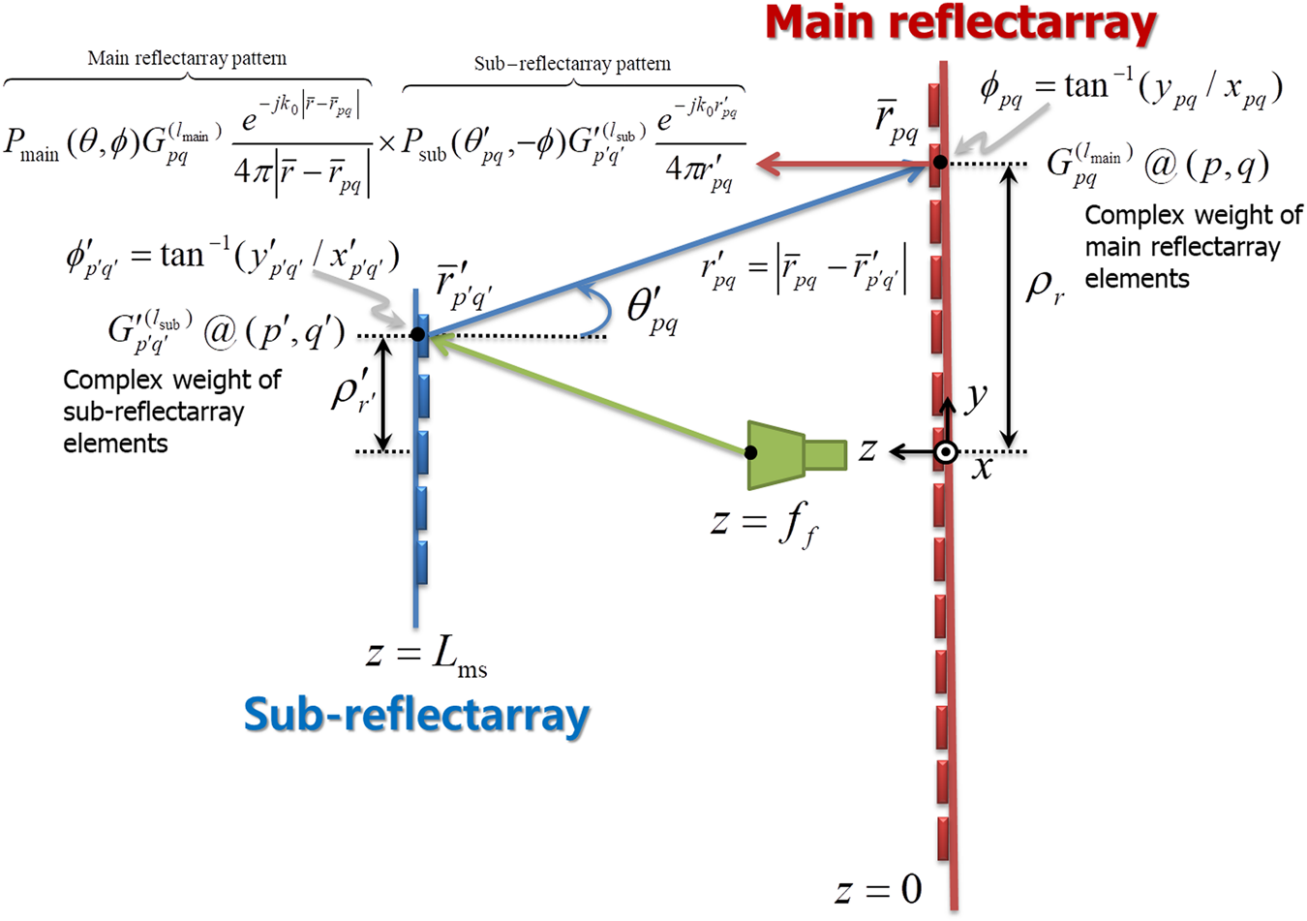
OAM mode-combining mechanism of a Cassegrain dual-reflectarray configuration composed of main and sub-reflectarrays excited by a feed. We set the complex weights of the main and sub-reflectarray elements at the array positions, (*p*,*q*) and $(p^{\prime} ,q^{\prime} )$, to ${G}_{pq}^{({l}_{{\rm{main}}})}={e}^{j{\psi }_{pq}^{{\rm{main}}}}$ and ${G}_{p^{\prime} q^{\prime} }^{\text{'}({l}_{{\rm{sub}}})}={e}^{j{\psi }_{p^{\prime} q^{\prime} }^{{\rm{sub}}}}$, respectively, where ${\psi }_{pq}^{{\rm{main}}}={\psi }_{0}^{{\rm{main}}}({\rho }_{pq})+{l}_{{\rm{main}}}{\varphi }_{pq}$, ${\psi }_{p^{\prime} q^{\prime} }^{{\rm{sub}}}={\psi }_{0}^{{\rm{sub}}}({\rho ^{\prime} }_{p^{\prime} q^{\prime} })+{l}_{{\rm{sub}}}{\varphi ^{\prime} }_{p^{\prime} q^{\prime} }$, and ${\psi }_{pq}^{{\rm{main}}}$, ${\psi }_{p^{\prime} q^{\prime} }^{{\rm{sub}}}$ are defined in the Supplementary Derivation of Equations (1) and (2).

To theoretically investigate the OAM mode-combining mechanism, we considered a simplified setup for the Cassegrain dual-reflectarray configuration, as illustrated in Fig. 2. Based on a conventional theory of array antennas^15^ and three-dimensional Green’s function identity in a free space [see the Supplementary Derivation of Equations (1) and (2)], the EM fields generated from the flat reflectarrays shown in Fig. 2 can be represented by the product of the element radiation pattern $P(\theta ,\varphi )$ and the array factor. When all reflective elements have the same independent radiation patterns, the resulting electric fields with *M* × *N* array elements are precisely formulated using the array factor. The flat reflectarray antennas shown in Fig. 1 are usually designed using *M* = *N* and the same spatial period, which represents a square reflectarray antenna. The reflected E-fields are thus given by

2 $${E}^{M\times M}(\bar{r})=-\frac{j{k}_{0}}{4\pi }\sum _{m=-{\rm{\infty }}}^{{\rm{\infty }}}{e}^{jm\varphi }\sum _{r=0}^{R-1}{E}_{m}(r,\theta ;{\rho }_{r}){e}^{j{\psi }_{0}({\rho }_{r})}\sum _{p,q}{e}^{j(l-m){\varphi }_{pq}},$$

where we set a complex weight ${G}_{pq}^{(l)}$ for the array element to ${e}^{j{\psi }_{pq}}$ in Equation (1); *p* and *q* are selected based on the condition of a constant radius, ${\rho }_{pq}={\rho }_{r}$; and the other parameters are as defined in the Supplementary Derivation of Equations (1) and (2). It should be noted that the dominant $l$th OAM mode can be formed using a square reflectarray with *m* = 1 and equally spaced ${\varphi }_{pq}$. This is because the accumulated phase terms [${e}^{j(l-m){\varphi }_{pq}}$] are canceled out based on the property of the discrete Fourier transform unless $(l-m){\varphi }_{pq}$ is a multiple of 2*π*. In the next step, we designed a Cassegrain dual-reflectarray antenna^16,17^ composed of main and sub-reflectarrays placed at *z* = 0 and ${L}_{ms}$, respectively, as shown in Fig. 2. Electromagnetic waves generated from a feed at $z={f}_{f}$ are reflected by a sub-reflectarray with complex weight ${G^{\prime} }_{{p}^{^{\prime} }{q}^{^{\prime} }}^{({l}_{{\rm{s}}{\rm{u}}{\rm{b}}})}={e}^{j{\psi }_{{p}^{^{\prime} }{q}^{^{\prime} }}^{{\rm{s}}{\rm{u}}{\rm{b}}}}$ of the hyperbolic phase shift. The reflected waves are subsequently transmitted to and re-radiated from the main reflectarray with ${G}_{pq}^{({l}_{{\rm{main}}})}={e}^{j{\psi }_{pq}^{{\rm{main}}}}$ of the parabolic phase shift. As a result, the total radiated E-fields from the Cassegrain dual-reflectarray antenna shown in Fig. 2 are represented by

3 $$\begin{array}{c}{E}^{{\rm{cass}}}(\bar{r})=-{(\frac{{k}_{0}}{4\pi })}^{2}\sum _{m=-\infty }^{\infty }{e}^{jm\varphi }\sum _{r}{E}_{m}^{{\rm{main}}}(r,\theta ;{\rho }_{r}){e}^{j{\psi }_{0}^{{\rm{main}}}({\rho }_{r})}\sum _{m^{\prime} =-\infty }^{\infty }\sum _{p,q}{e}^{j({l}_{{\rm{main}}}+m^{\prime} -m){\varphi }_{pq}}\\ \quad \quad \quad \quad \,\times \sum _{r^{\prime} }{E}_{-m^{\prime} }^{{\rm{sub}}}({r}_{pq}^{^{\prime} },{\theta }_{pq}^{^{\prime} };{\rho }_{r^{\prime} }^{^{\prime} }){e}^{j{\psi }_{0}^{{\rm{sub}}}({\rho }_{r^{\prime} }^{^{\prime} })}\sum _{p^{\prime} ,q^{\prime} }{e}^{j({l}_{{\rm{sub}}}+m^{\prime} ){\varphi }_{p^{\prime} q^{\prime} }^{^{\prime} }}\mathrm{.}\end{array}$$

Here, ${\varphi }_{pq}$ and ${\varphi }_{p^{\prime} q^{\prime} }^{^{\prime} }$ are progressively changed along the azimuth for the given ${\rho }_{r}$ and ${\rho }_{r^{\prime} }^{^{\prime} }$, respectively. According to Equations (2) and (3), we conclude that the dominant total OAM mode number of the Cassegrain dual-reflectarray antenna is arithmetically combined, the reason for which is that only $m={l}_{{\rm{main}}}+m^{\prime}$ and $m^{\prime} =-{l}_{{\rm{sub}}}$ remain un-canceled owing to Equation (2). Therefore, EM waves doubly-reflected by the reflectarrays show the combined total OAM mode number ${l}_{{\rm{tot}}}$ as

4 $${l}_{{\rm{tot}}}={l}_{{\rm{main}}}-{l}_{{\rm{sub}}},$$

where the minus sign indicates that the main and sub-reflectarrays face each other.

**Figure 3:**
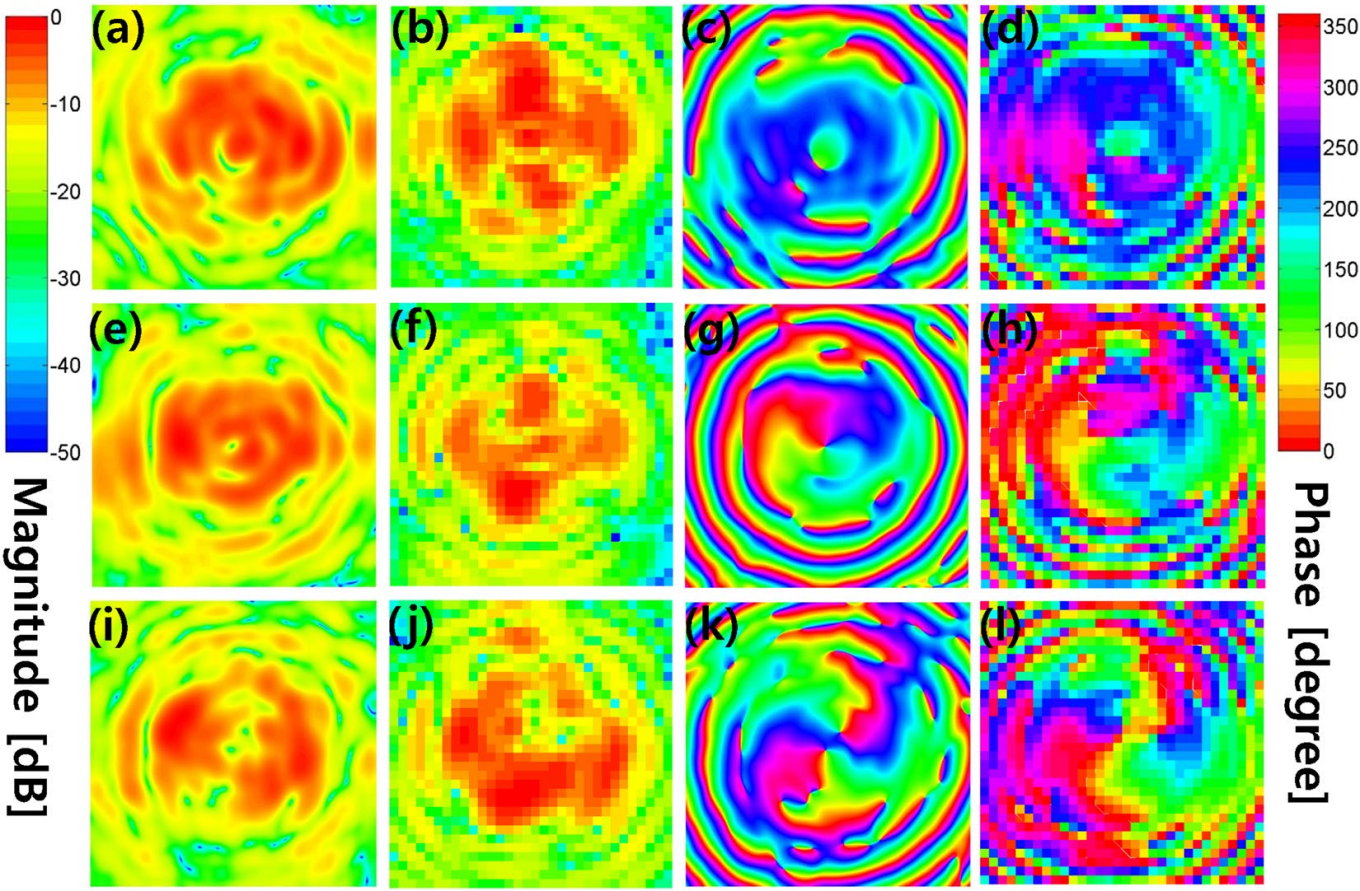
Near-field magnitude and phase patterns of the Cassegrain dual-reflectarray antennas in Fig. 1 simulated and measured using *f* = 18 [GHz], *z* = 124 [mm], and *l*_main_ = +1. The main reflectarray is placed at *z* = 0. **(a,e,i)** Simulated magnitude patterns for ${l}_{{\rm{sub}}}=+1,\,0,\,-1$, respectively. **(b,f,j)** Measured magnitude patterns for ${l}_{{\rm{sub}}}=+1,0,-1$, respectively. **(c,g,k)** Simulated phase patterns for ${l}_{{\rm{sub}}}=+\mathrm{1,\; 0,}\,-1$, respectively. **(d,h,l)** Measured phase patterns for ${l}_{{\rm{sub}}}=+\mathrm{1,\; 0},-1$, respectively.

**Figure 4:**
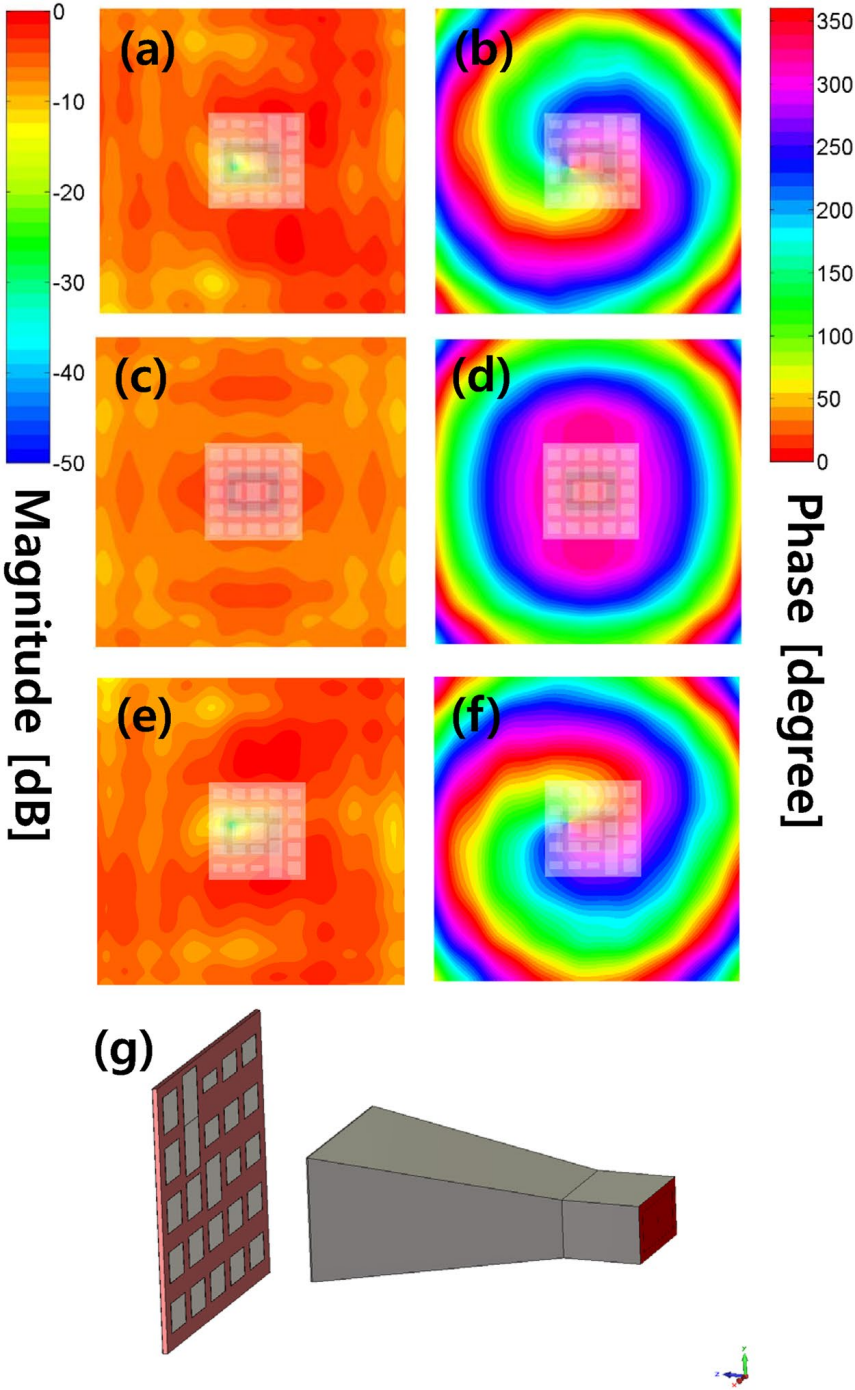
Simulated near-field magnitude and phase patterns using *f* = 18 [GHz] and *z* = −100 [mm] for the Cassegrain dual-reflectarray antenna without the main reflectarray shown in Fig. 1. The edges of the horn aperture and sub-reflectarray are placed at *z* = 23.6 and 44.4 [mm], respectively. The sub-reflectarrays are overlaid with the near-field patterns to clarify helical phase centers of the given OAM mode numbers, where a helical phase center means a point satisfying the minimum magnitude in term of helical phase. We observe that the corresponding $(x,y)$ positions of the helical phase centers vary according to ${l}_{{\rm{sub}}}=\pm 1$. **(a,c,e)** Simulated magnitude patterns for ${l}_{{\rm{sub}}}=+1,0,-1$, respectively. **(b,d,f)** Simulated phase patterns for ${l}_{{\rm{sub}}}=+1,0,-1$, respectively. **(g)** Example of a simulation setup for ${l}_{{\rm{sub}}}=+1$.

**Figure 5:**
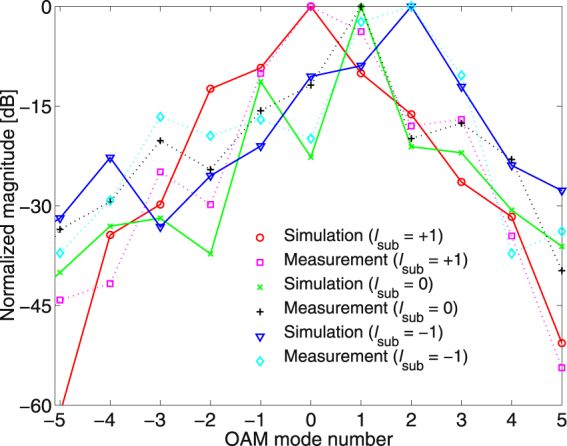
Simulated and measured normalized OAM modal magnitudes versus OAM mode numbers in the far-field at 18 [GHz]. The normalized modal magnitudes for ${l}_{{\rm{sub}}}=+1,0,-1$ were simulated and measured at $\theta ={4}^{\circ },\,{5}^{\circ },\,{6}^{\circ }$, respectively, when ${l}_{{\rm{main}}}=+1$. The near-field distributions in Fig. 3 were first measured in the planar near-field range and then transformed to obtain the far-field azimuthal patterns and normalized modal magnitudes of the corresponding OAM mode numbers.

**Figure 6:**
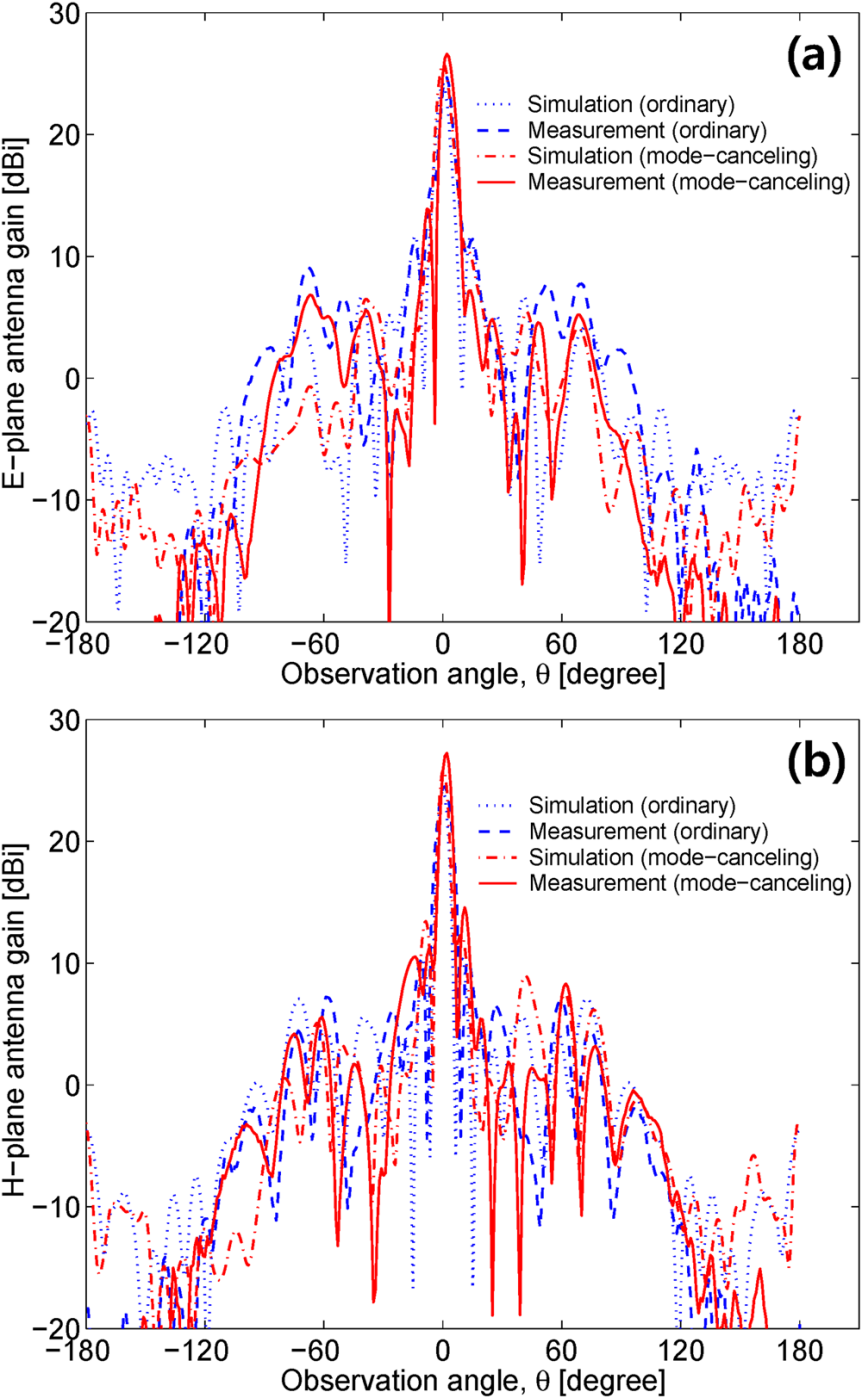
Simulated and measured antenna gain patterns of ordinary (${l}_{{\rm{main}}}={l}_{{\rm{sub}}}=0$) and mode-canceling (*l*_main_ = *l*_sub_ = + 1) reflectarray antennas composed of microstrip patches on the main and sub-reflectarrays at 18 [GHz]. Radiation patterns and antenna gains were measured in the far-field range. **(a)** E-plane ($\varphi ={90}^{\circ }$) antenna gain. **(b)** H-plane ($\varphi ={0}^{\circ }$) antenna gain.

Figure 3 shows the simulated and measured near-field magnitude and phase distributions of the Cassegrain dual-reflectarrays with ${l}_{{\rm{sub}}}=0,\pm 1$, when the OAM mode number of the main reflectarray is fixed at ${l}_{{\rm{main}}}=+1$. In Fig. 3, the distinct OAM mode-combined patterns indicate that the OAM mode numbers shift from ${l}_{{\rm{sub}}}=+1,0,-1$ to ${l}_{{\rm{tot}}}=0,+1,+2$, as expected from Equation (4). Figure 4 also shows the near-field patterns of ${l}_{{\rm{sub}}}=+\mathrm{1,\; 0,}-1$ generated only by the sub-reflectarray and horn feed. Microstrip patch elements on the sub-reflectarray are configured by Equation (1) to reflect the desired OAM mode *l*_sub_. In the next step, the near-fields in Fig. 4 are again reflected by the main reflectarray with ${l}_{{\rm{main}}}=+1$, and the resultant field distributions are then as illustrated in Fig. 3. Based on the near-field to far-field transformed radiation patterns (see Figs 6 and 7 in the Supplement for the corresponding far-field patterns), the normalized OAM modal magnitudes in the far-field radiated from the mode-combined reflectarrays are as shown in Fig. 5. A normalized OAM modal magnitude, which also represents the OAM mode purity in the far-field, is defined as $|{F}_{m}^{(l)}(\theta )/{F}_{l}^{(l)}(\theta )|$, where a far-field radiation pattern is expanded by ${E}^{{\rm{far}}}(\theta ,\varphi )={\sum }_{m=-\infty }^{\infty }{F}_{m}^{(l)}(\theta ){e}^{jm\varphi }$. As shown in Figs 3 and 5, the mode-combined OAM states (${l}_{{\rm{tot}}}$) in the near-field are continuously conserved in the far-field, thereby confirming that Equation (4) is satisfied even in the far-field including the near-field. Consequently, Figs 3 and 5 indicate that the OAM modes of the EM fields can be arithmetically added or subtracted in both the near- and far-fields. The OAM mode-combining phenomenon also exists in a Cassegrain dual-reflector configuration (see the Supplementary Measurement of a Cassegrain Dual-reflector Antenna). When ${l}_{{\rm{main}}}=-1$ and ${l}_{{\rm{sub}}}=+1$ are selected for the helicoidal surfaces on the main and subreflectors, respectively, the simulated and measured near- and far-field results justify the total OAM mode number being combined as ${l}_{{\rm{tot}}}=-2$ according to Equation (4). In addition, the quality of the azimuthal phase variation of helicoidal reflectors is better than that of microstrip patch reflectarrays owing to the continuously generated helical phase.

### OAM Mode-canceling Antennas

**Figure 7:**
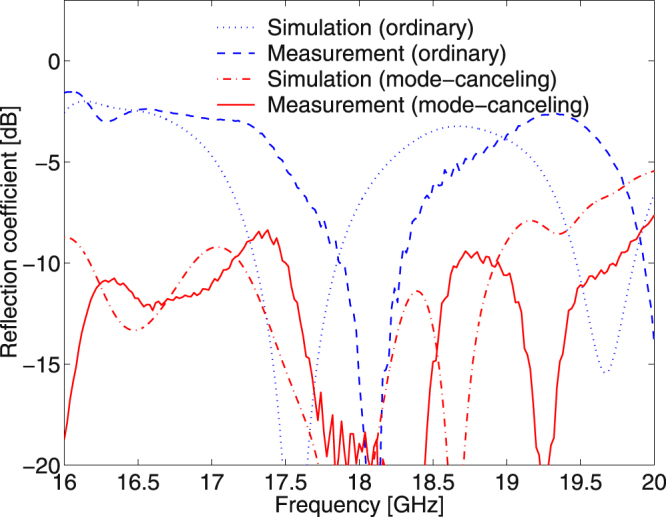
Simulated and measured reflection coefficients (*S*_11_) of ordinary (${l}_{{\rm{main}}}={l}_{{\rm{sub}}}=0$) and mode-canceling (*l*_main_ = *l*_sub_ = + 1) reflectarray antennas versus frequency. The −10dB reflection bandwidth is also given in Table 1.

**Figure 8:**
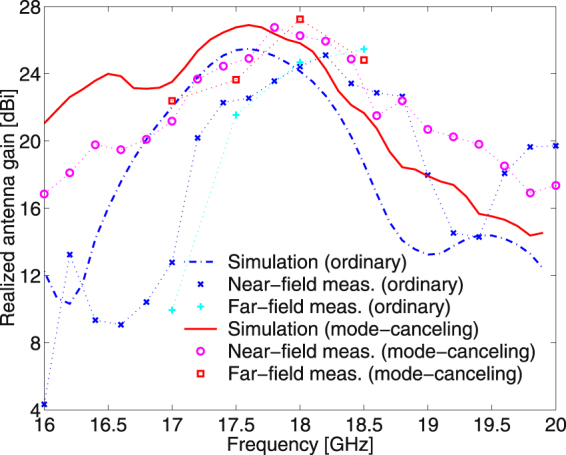
Simulated and measured realized antenna gains of ordinary (${l}_{{\rm{main}}}={l}_{{\rm{sub}}}=0$) and mode-canceling (*l*_main_ = *l*_sub_ = + 1) reflectarray antennas versus frequency. The antenna gains were measured in the near- and far-field ranges. In addition, the maximum realized gain and 3 dB realized gain bandwidth are shown in Table 1.

Far-field radiation patterns of non-zero OAM modes theoretically show zero magnitude at *θ* = 0°. This is because the non-zero OAM modes do not radiate around *θ* = 0° owing to a helical phase cancelation. Hence, OAM mode-generating antennas generally have a severe weakness with high-gain applications. When the OAM mode numbers of the main and sub-reflectarrays shown in Fig. 1 are the same, which is ${l}_{{\rm{main}}}={l}_{{\rm{sub}}}$ in Equation (4), the mode-combined EM waves arithmetically cancel out the opposite phase behaviors in the near-field, and thus the main beam pattern of that antenna is similar to that of an ordinary high-gain antenna in the far-field. Because we select the same OAM mode numbers for the main and sub-reflectarrays in Fig. 1 as ${l}_{{\rm{main}}}={l}_{{\rm{sub}}}=+1$, our Cassegrain dual-reflectarray configuration becomes an OAM mode-canceling antenna (MCA). Figure 6 shows the far-field radiation patterns of ordinary (${l}_{{\rm{main}}}={l}_{{\rm{sub}}}=0$) and mode-canceling (${l}_{{\rm{main}}}={l}_{{\rm{sub}}}=+1$) reflectarray antennas. Radiation measurements were conducted in the far-field range, as illustrated in Fig. 1b. In Fig. 6, we can see that the OAM mode-canceling beams radiated by the MCA do not have zero magnitude near *θ* = 0° and the main beam pattern is bell-shaped as with an ordinary high-gain antenna. This is because the ${l}_{{\rm{sub}}}=+1$ OAM wave produced by the sub-reflectarray, whose central magnitude near *θ* = 180° illustrated in Fig. 4(a) is very small, is combined and subsequently compensated using the adjacent main reflectarray with ${l}_{{\rm{main}}}=+1$. As a result, part with zero magnitude in the far-field is restored by the OAM mode cancelation in the near-field. Table 1 indicates that the maximum realized antenna gains of the proposed MCA are 1.7, 2.5, and 1.9 dB higher than those of the ordinary reflectarray antenna in terms of the simulation, far-field, and near-field measurements, respectively. The −10dB reflection bandwidth of the MCA also shows a better performance than that of an ordinary reflectarray, whereas the MCA does not improve the 3 dB realized gain bandwidth. The reflection coefficients (*S*_11_) of the ordinary and mode-canceling reflectarray antennas are given in Fig. 7. The *S*_11_ results are measured at the end of the horn feeds, as shown in Fig. 1. Figures 6 and 7 indicate that the radiation and reflection characteristics of the MCA are better than those of the ordinary reflectarray antenna, thus confirming that OAM mode canceling can be used to design a new type of low-profile, broad-reflection bandwidth, and high-gain reflector antenna. The frequency characteristics versus the realized antenna gains are illustrated in Fig. 8. When the focal-length-to-diameter ratio (*F*/*D*) of a Cassegrain reflector antenna is quite small, the reflected waves directly from a Cassegrain (hyperbolic) subreflector make the reflection characteristics worse, thereby resulting in the decrease of the realized antenna gain. However, it is clear that the sub-reflectarray with non-zero OAM modes ($l\ne 0$) and the ordinary horn feed (*l* = 0) are inherently decoupled because the isolation levels among the different OAM modes are relatively high^8,14,19^. Therefore, based on the simulation and measurement results, the realized gain of the MCA is always higher than that of the ordinary reflectarray when the frequency is below 18.2 [GHz]. This is mainly attributed to the much better reflection coefficients caused by the OAM mode isolation, as previously shown in Fig. 7.

**Table 1:** Comparison of maximum realized antenna gain [dBi], −10dB reflection (*S*_11_) bandwidth [GHz], and 3 dB realized gain bandwidth [GHz] of the ordinary (*l*_main_ = *l*_sub_ = 0) and mode-canceling (*l*_main_ = *l*_sub_ = + 1) reflectarray antennas. The realized antenna gains were computed and measured at 18 [GHz]. The reflection coefficients were measured using a network analyzer (Anritsu 37397 C).

Antenna	Type	Simulation	Far-field range	Near-field range
Ordinary	Realized gain	24.2	24.7	24.4
	*S*_11_ bandwidth	0.55	—	0.41
	Gain bandwidth	1.17	—	1.44
Mode-canceling	Realized gain	25.9	27.2	26.3
	*S*_11_ bandwidth	1.73	—	1.18
	Gain bandwidth	1.18	—	1.24

## Discussion

We fabricated the Cassegrain dual-reflectarray and dual-reflector antennas with the microstrip patches shown in Fig. 1, as well as helicoidal reflectors (see the Supplementary Measurement of a Cassegrain Dual-reflector Antenna). Measurements of both the reflectarrays and reflectors show that the OAM modes are arithmetically combined or canceled in the near- and far-fields according to the sign of the OAM mode numbers formed on the closely placed reflectarrays and reflectors. The MCA with ${l}_{{\rm{main}}}={l}_{{\rm{sub}}}=+1$ also shows excellent radiation and reflection characteristics, as indicated in Figs 6–8 and Table 1. The concept of an MCA is a new solution for high-gain reflectarray and reflector antennas, in which the main reflectarray and reflector usually have a large focal-length-to-diameter ratio (*F*/*D*) or high antenna profile to maintain the direct reflection to the feeds as low as possible. When the sub-reflectarray and subreflector re-radiate non-zero OAM modes ($l\ne 0$), the received power into the conventional horn feeds ($l\mathrm{=0}$) is quite small. Therefore, the distance between a sub-reflectarray/subreflector and feed in the proposed MCA can be shorter than that in an ordinary antenna, whereas the reflection coefficients (*S*_11_) have the same level for both cases. In view of a traditional Cassegrain configuration^15,16^, a mutual coupling effect of the main and sub-reflectarrays in Fig. 1 is insignificant because the EM waves reflected by the sub-reflectarray are obliquely incident to the main reflectarray and strongly collimated to the zenith direction, $\theta ={0}^{\circ }$ (see Fig. 4 in the Supplement). On the contrary, the severe interaction usually occurs between a sub-reflectarray and feed. The MCA can alleviate this mutual coupling owing to the OAM mode isolation that the reflection coefficient (*S*_11_) caused by the *l*_sub_ = ±1 is much smaller than that by the *l*_sub_ = 0 (see Fig. 8 in the Supplement).

## Methods

Cassegrain dual-reflectarray antennas with the main and sub-reflectarrays shown in Fig. 1 were fabricated on dielectric substrates with an ${\varepsilon }_{r}$ of 2.2 and thickness of 0.7874 [mm] (see the Supplementary Measurement of Cassegrain Dual-reflectarray Antennas for detailed antenna parameters). The period and width of all microstrip patch elements are 8.3 and 5.94 [mm], respectively. The length of each patch is controlled to generate a specific OAM mode radiation. Equation (1) was used to obtain the initial design parameters of those reflectarrays, and the final Cassegrain configurations were then simulated using CST Microwave Studio. The Cassegrain dual-reflectarray antennas were measured at 18 [GHz] in the near- and far-field ranges. We used a planar near-field antenna measurement method with a scan area of 528 [mm] × 528 [mm] and sample spacing of 8 [mm]. The E- ($\theta ={90}^{\circ }$) and H-plane ($\theta ={0}^{\circ }$) far-field measurements were conducted in an anechoic chamber, the dimensions of which are 7 [m] wide, 14 [m] long, and 6.7 [m] high.

### Data Availability

The simulation and measurement datasets generated during and/or analysed during the current study are available from the corresponding author on reasonable request.

## Electronic supplementary material

Supplementary Info
