# Supplementary material for: Orbital Angular Momentum (OAM) Antennas via Mode Combining and Canceling in Near-field
Source: Sci Rep. 2017 Oct 9;7:12805. doi: 10.1038/s41598-017-13125-5 (PMC5634501; doi:10.1038/s41598-017-13125-5)
Supplement: Supplementary file 1 — Supplementary Info [file 41598_2017_13125_MOESM1_ESM.pdf]

Orbital Angular Momentum (OAM) Antennas via Mode Combining and Canceling in Near-field

Woo Jin Byun*, Hyung Do Choi†, and Yong Heui Cho‡
*Radio Resource Research Group, Electronics and Telecommunications Research Institute (ETRI), Daejeon, 34129, Korea
†Radio Environment & Monitoring Research Group, Electronics and Telecommunications Research Institute (ETRI), Daejeon, 34129, Korea
‡School of Information and Communication Engineering, Mokwon University, Daejeon, 35349, Korea
E-mail: yongheui.cho@gmail.com

I. Supplementary Derivation of Equations (1) and (2)

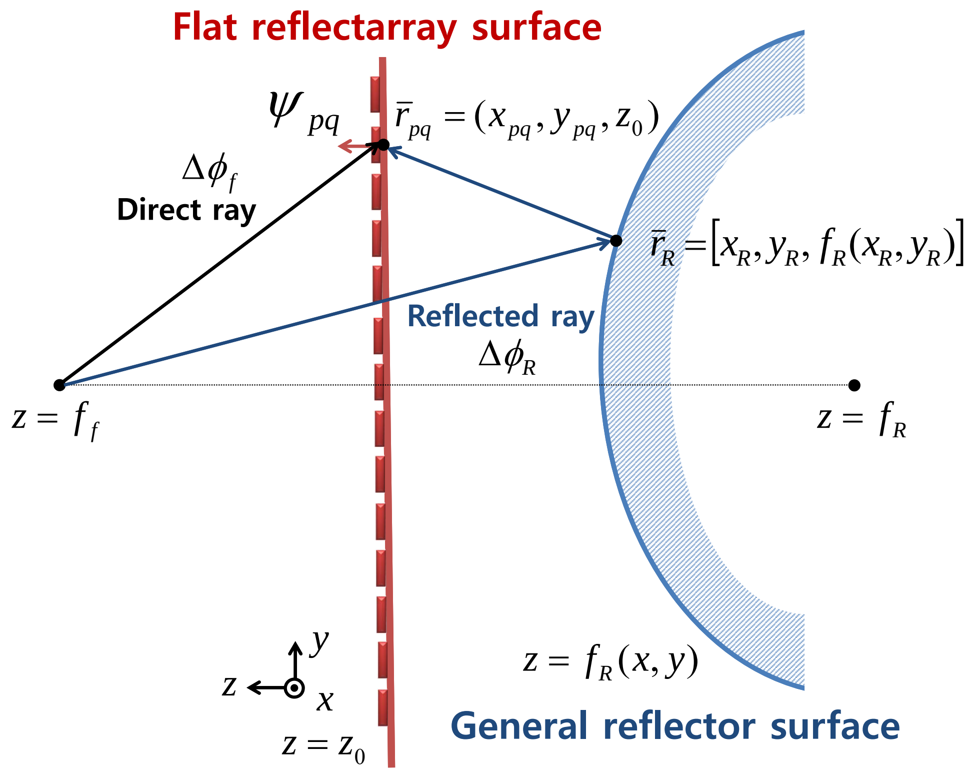

Fig. 1. Reflection phase formation of a flat reflectarray surface based on the geometric phase equivalence to a general reflector surface.

Based on the concept of a reflectarray metasurface [1], [2], a flat reflectarray surface shown in Fig. 1 re-radiates arbitrary reflective beams by changing the physical dimensions of each array element. Consider a general reflector surface in Fig. 1 whose equation is represented as $z = f_R(x, y)$. Equating that the reflection phases from the flat reflectarray surface are geometrically equivalent to those from the general reflector surface, we obtain a phase-matching condition ψ_{pq} for the (p, q)th array element in the targeting reflectarray as

$$\Delta\phi_f + \psi_{pq} = \Delta\phi_R \ \Rightarrow \ \psi_{pq} = \Delta\phi_R - \Delta\phi_f \ , \tag{1}$$

where the (p, q)th element is placed at $\bar{r}_{pq}$, f_f and f_R are the phase center and focus of a feed and reflector, respectively,

$$\Delta\phi_f = -k_0|\bar{r}_{pq} - (0, 0, f_f)| \tag{2}$$

$$\Delta\phi_R = -k_0\left[|\bar{r}_R - (0, 0, f_f)| + |\bar{r}_{pq} - \bar{r}_R|\right] \ , \tag{3}$$

and k_0 is a free-space wavenumber. It is noted that the time convention is selected as $e^{j\omega t}$. By adding a vortex phase term $(l\phi)$ for the l-times phase rotation, the phase-matching condition ψ_{pq} in (1) is also used to design a reflectarray antenna for the lth orbital angular momentum (OAM) mode as

$$\psi_{pq} = \Delta\phi_R - \Delta\phi_f + l\phi_{pq} = \psi_0(\rho_{pq}) + l\phi_{pq} \ , \tag{4}$$

where $\rho_{pq} = \sqrt{x_{pq}^2 + y_{pq}^2}$ and $\phi_{pq} = \tan^{-1}(y_{pq}/x_{pq})$. When the reflector surfaces $f_R(x, y)$ represent a parabolic main reflector and a Cassegrain (hyperbolic) subreflector, (4) reduces to the corresponding design formulas as, respectively,

$$\psi_{pq}^{\text{main}} = k_0\left[\sqrt{\rho_{pq}^2 + (f_f - z_0)^2} - f_f - z_0 + 2f_R(0,0)\right] + l\phi_{pq} \tag{5}$$

$$\psi_{pq}^{\text{sub}} = k_0\left[\sqrt{\rho_{pq}^2 + (f_f - z_0)^2} - \sqrt{\rho_{pq}^2 + (z_0 - f_R)^2}\right] + l\phi_{pq} \ . \tag{6}$$

Similarly, the OAM state-forming relation (4) can be applied to any kind of general reflector surfaces including the Gregorian and axially displaced ellipse (ADE) subreflectors.

In terms of an array antenna [3], the electromagnetic fields generated from the flat reflectarray shown in Fig. 1 can be represented by the product of the element radiation pattern $P(\theta,\phi)$ and the array factor. When all reflective elements have the same independent radiation patterns, the resulting electric fields with $M \times N$ elements are formulated as

$$E^{M\times N}(\bar{r}) \;=\; \sum_{p=0}^{M-1}\sum_{q=0}^{N-1} G_{pq}^{(l)} \frac{e^{-jk_0|\bar{r}-\bar{r}_{pq}|}}{4\pi\,|\bar{r}-\bar{r}_{pq}|} \;, \tag{7}$$

where we omitted $P(\theta,\phi)$ for brevity, $\bar{r}=(x,y,z)$, $\bar{r}_{pq}=(x_{pq},y_{pq},z_0)$, and $G_{pq}^{(l)}$ is a complex weight of the (p,q)th array element to form the lth OAM mode. Utilizing three-dimensional Green's function identity in free space,

$$\frac{e^{-jk_0|\bar{r}-\bar{r}'|}}{4\pi\,|\bar{r}-\bar{r}'|} \;=\; -jk_0 \begin{cases} \displaystyle\sum_{n=0}^{\infty}\sum_{m=-n}^{n} j_n(k_0 r') h_n^{(2)}(k_0 r) Y_n^m(\theta,\phi)\big[Y_n^m(\theta',\phi')\big]^* & \text{for } r > r' \\ \displaystyle\sum_{n=0}^{\infty}\sum_{m=-n}^{n} j_n(k_0 r) h_n^{(2)}(k_0 r') Y_n^m(\theta,\phi)\big[Y_n^m(\theta',\phi')\big]^* & \text{for } r < r' \end{cases} \;, \tag{8}$$

we reformulate (7) in the same spherical coordinate system as

$$E^{M\times N}(\bar{r}) \;=\; -\frac{jk_0}{4\pi}\sum_{m=-\infty}^{\infty} e^{jm\phi} \sum_{p=0}^{M-1}\sum_{q=0}^{N-1} E_m(r,\theta;\rho_{pq}) G_{pq}^{(l)} e^{-jm\phi_{pq}} \;, \tag{9}$$

where $r=|\bar{r}|$, $\theta=\cos^{-1}(z/r)$, $\phi=\tan^{-1}(y/x)$, $z_0=0$,

$$E_m(r,\theta;\rho') \;=\; j^{-m}\sum_{n=|m|}^{\infty} j^{-n}(2n+1)\frac{(n-m-1)!!}{(n+m)!!} j_n(k_0\rho') h_n^{(2)}(k_0 r) P_n^m(\cos\theta)\Bigg|_{n+m\text{: even}} \;, \tag{10}$$

$j_n(\cdot)$ and $h_n^{(2)}(\cdot)$ are the nth-order spherical Bessel and Hankel functions of the first and second kinds, respectively, $(\cdot)!!$ is a double factorial, and $Y_n^m(\theta,\phi)$ are the spherical harmonics composed of the complex exponentials and associated Legendre polynomials as

$$Y_n^m(\theta,\phi) \;=\; \sqrt{\frac{2n+1}{4\pi}\frac{(n-m)!}{(n+m)!}} P_n^m(\cos\theta) e^{jm\phi} \;. \tag{11}$$

In the far-field region, (9) further simplifies to

$$E^{M\times N}(\bar{r}) \;\sim\; \frac{e^{-jk_0 r}}{4\pi r}\sum_{m=-\infty}^{\infty} e^{jm\phi} \sum_{p=0}^{M-1}\sum_{q=0}^{N-1} \mathrm{AF}_m(\theta;\rho_{pq}) G_{pq}^{(l)} e^{-jm\phi_{pq}} \;, \tag{12}$$

where

$$\mathrm{AF}_m(\theta;\rho') \;=\; j^{-m}\sum_{n=|m|}^{\infty} (2n+1)\frac{(n-m-1)!!}{(n+m)!!} j_n(k_0\rho') P_n^m(\cos\theta)\Bigg|_{n+m\text{: even}} \;. \tag{13}$$

When the array elements are arranged with a uniform circular array (UCA) [4] configuration [$\rho_{pq}=\rho_0$, $\phi_{pq}=(pN+q)\Delta\phi$, $\Delta\phi=\frac{2\pi}{MN}$] and $G_{pq}^{(l)}=e^{jl\phi_{pq}}$, (9) results in the OAM mode selection as

$$\begin{aligned} E^{M\times N}(\bar{r}) \;&=\; -\frac{jk_0}{4\pi} MN \sum_{m=-\infty}^{\infty} E_{l+mMN}(r,\theta;\rho_0) e^{j(l+mMN)\phi} \\ &\approx\; -\frac{jk_0}{4\pi} MN \cdot E_l(r,\theta;\rho_0) e^{jl\phi} \quad \text{when } MN \gg 1 \;. \end{aligned} \tag{14}$$

Therefore, (14) indicates that the UCA with the predetermined incremental phase shift generates the dominant lth OAM mode, even though the higher-order OAM modes appear periodically with a MN spacing.

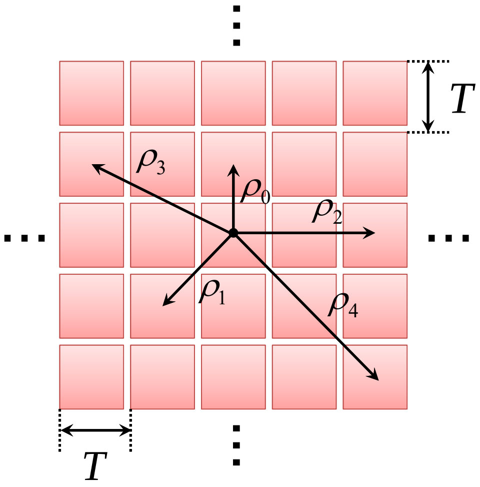

Fig. 2. Various radii ($\rho = \sqrt{x^2 + y^2}$) appeared in the periodic array elements shown in Fig. 1, when M and N are odd numbers.

The flat reflectarray antennas shown in Fig. 1 are usually designed with $M = N$ and the same spatial period (T) as illustrated in Fig. 2. Then, the reflected E-fields are given by

$$E^{M\times M}(\bar{r}) \;=\; -\frac{jk_0}{4\pi}\sum_{m=-\infty}^{\infty} e^{jm\phi}\sum_{r=0}^{R-1} E_m(r,\theta;\rho_r)e^{j\psi_0(\rho_r)}\sum_{p,q} e^{j(l-m)\phi_{pq}}\;, \tag{15}$$

where $G_{pq}^{(l)} = e^{j\psi_{pq}}$, $R = \frac{M^2+4M-5}{8}$, M is an odd number, $\psi_0(\rho_r)$ is defined in (4), and p, q are selected with the condition $\rho_{pq} = \rho_r$.

All phase terms in (5) and (6) can be synthesized using the one-to-one relationship between the reflected phase and microstrip patch length as shown in Fig. 3. This means that any phase shift for (5) and (6) is obtained by changing the patch length according to the design curve in Fig. 3.

Fig. 4 illustrates the simplified ray distributions of Cassegrain reflector and reflectarray antennas. In view of a Cassegrain configuration [3], [5], most rays launched from the Cassegrain subreflector and flat sub-reflectarray are obliquely incident to the parabolic main reflector and flat main reflectarray, respectively. The doubly reflected rays by the main reflector/reflectarray are in phase, thus yielding the high gain radiation usually to the zenith direction. Some of rays from the feed are also reflected by the adjacent subreflector/sub-reflectarray surfaces and return directly to the input feed. This direct reflection causes the higher reflection coefficients as the focal-length-to-diameter ratio (F/D) becomes smaller. Therefore, a mutual coupling between the subreflector/sub-reflectarray and main reflector/reflectarray is negligible except for the feed area.

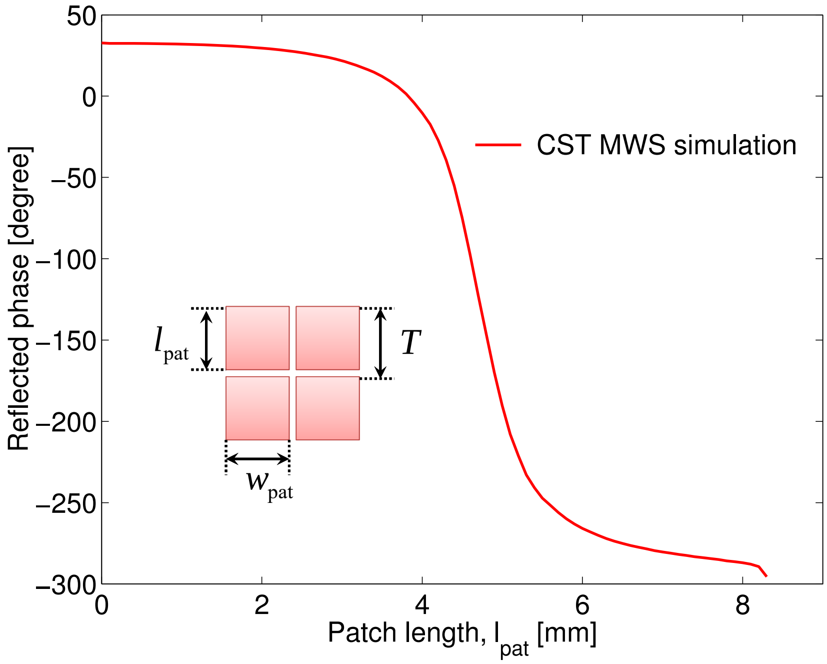

Fig. 3. Reflected phase versus microstrip patch length, $l_{\rm pat}$ when a uniform plane wave is incident normally on the reflectarray surface and the periodic boundary conditions are assumed. The design parameters are as follows: T = 8.3 [mm], $w_{\rm pat} = 5.94$ [mm], and a Taconic TLY-5 substrate ($\epsilon_r = 2.2$, thickness = 0.7874 [mm]). Numerical computations are conducted using CST Microwave Studio.

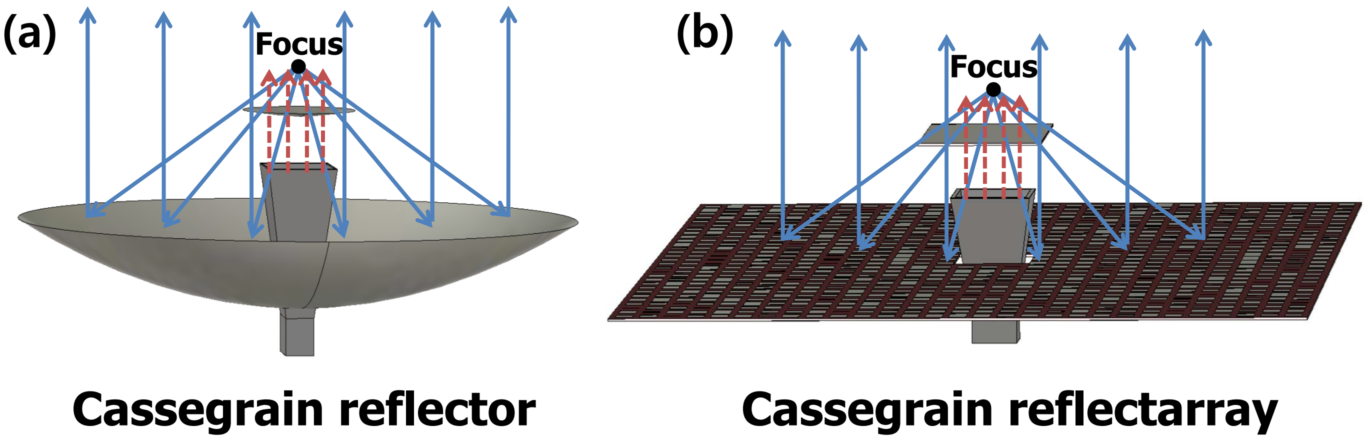

Fig. 4. Ray distributions of **(a)** Cassegrain reflector and **(b)** Cassegrain reflectarray antennas. The rays (blue solid lines) are equivalently launched from virtual foci formed by the Cassegrain (hyperbolic) subreflector and flat sub-reflectarray. The subreflector and sub-reflectarray are excited by ordinary horn feeds (red dashed lines).

II. Supplementary Measurement of Cassegrain Dual-reflectarray Antennas

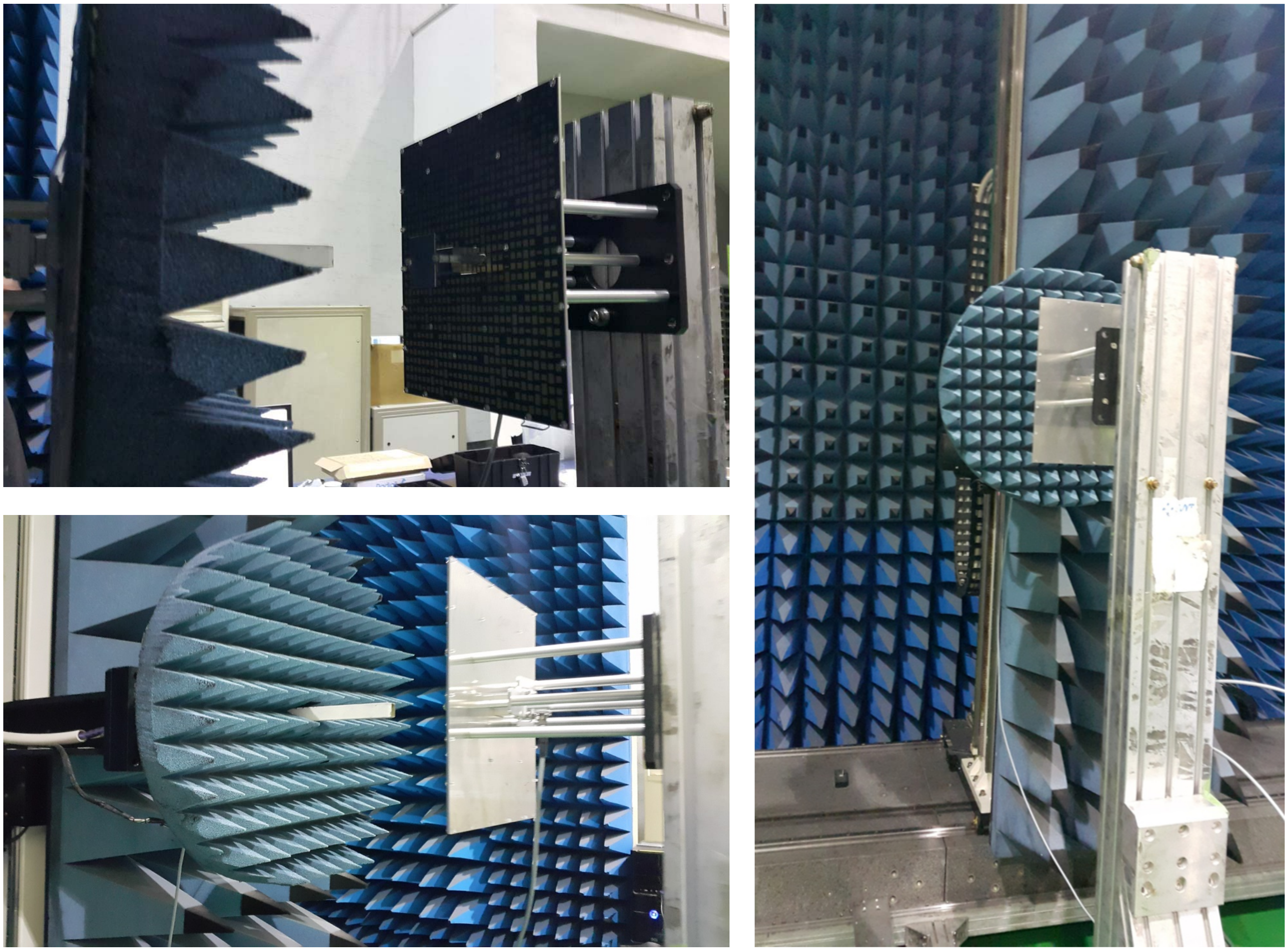

Fig. 5. Near-field antenna measurement setup with the attached fabricated Cassegrain dual-reflectarray antenna. Based on Eqn. (4) and [5], the Cassegrain dual-reflectarray antennas were designed and fabricated using the main ($l_{\text{main}} = +1$) and sub-reflectarrays ($l_{\text{sub}} = 0, \pm 1$) composed of multiple microstrip patches (period = 8.3 [mm] and width = 5.94 [mm]) on dielectric substrates (Taconic TLY-5). Primary design parameters are as follows: f_m/D_m = 0.4, D_s/D_m = 0.2, -10dB beamwidth of the horn feed = 90°, the width and length of the rectangular main reflectarray = 250 [mm], and a substrate ϵ_r = 2.2, and a substrate thickness = 0.7874 [mm], where f_m is the focus of the main reflectarray and D_m, D_s are the diameters of the main and sub-reflectarrays, respectively.

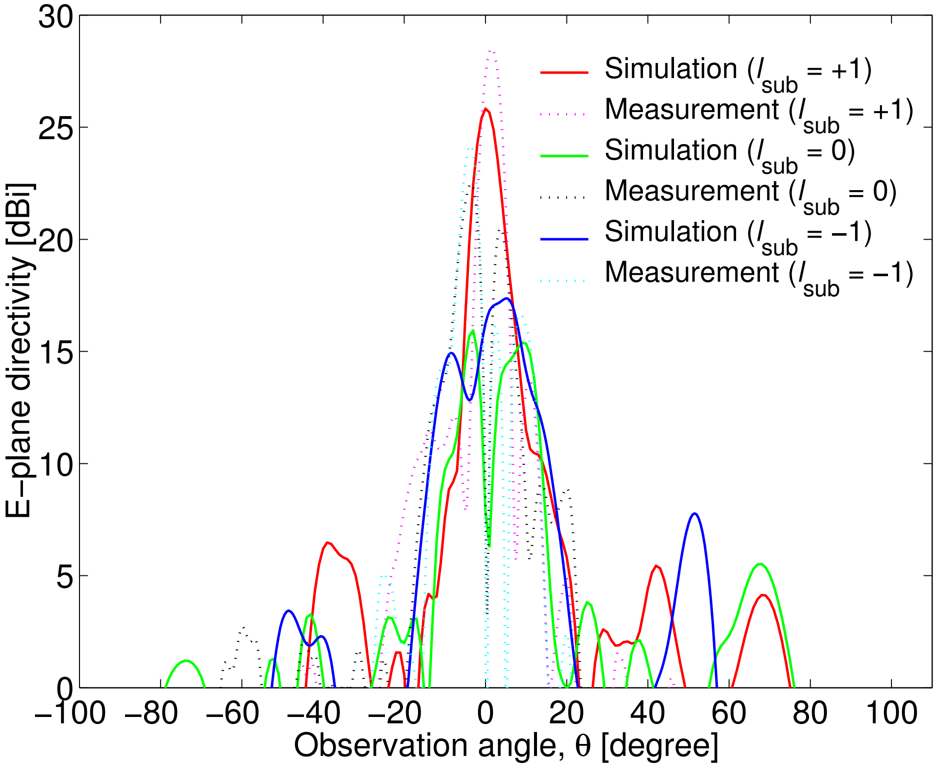

(a)

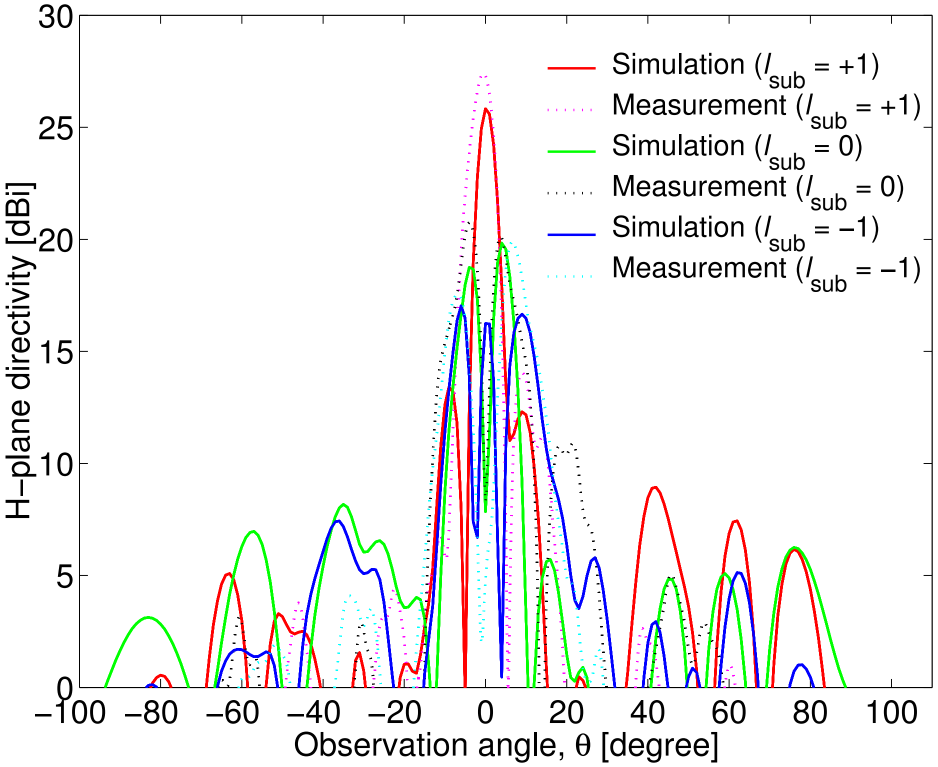

(b)

Fig. 6. Simulated and measured directivity patterns of the Cassegrain dual-reflectarray antennas illustrated in Fig. 5 using f = 18 [GHz] and $l_{\text{main}} = +1$. Measurement results are obtained by the measured near-field to far-field transformation. **(a)** E-plane ($\phi = 90^\circ$) directivity. **(b)** H-plane ($\phi = 0^\circ$) directivity.

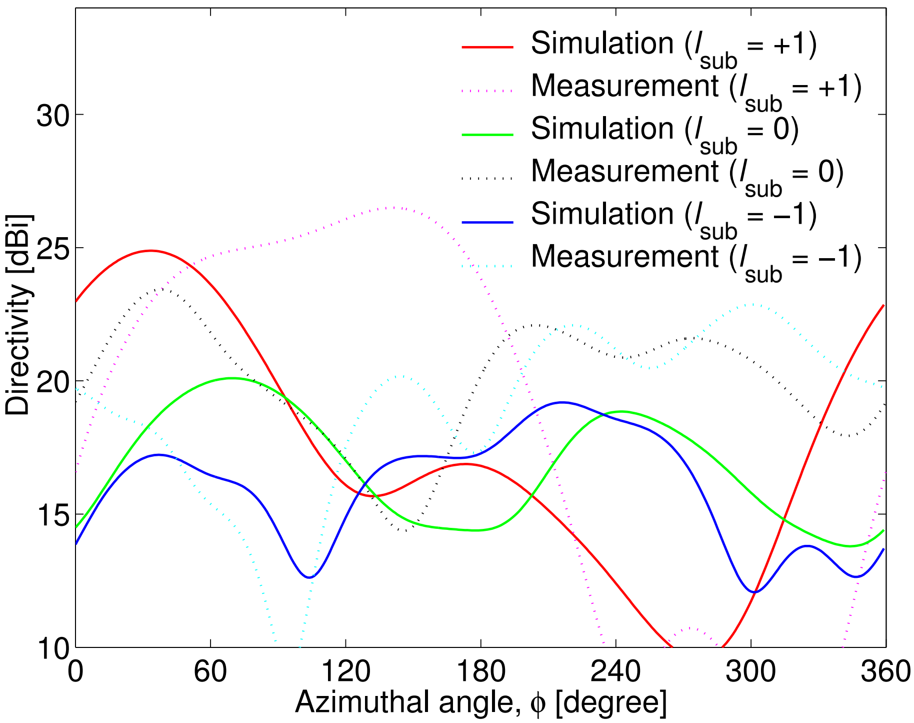

(a)

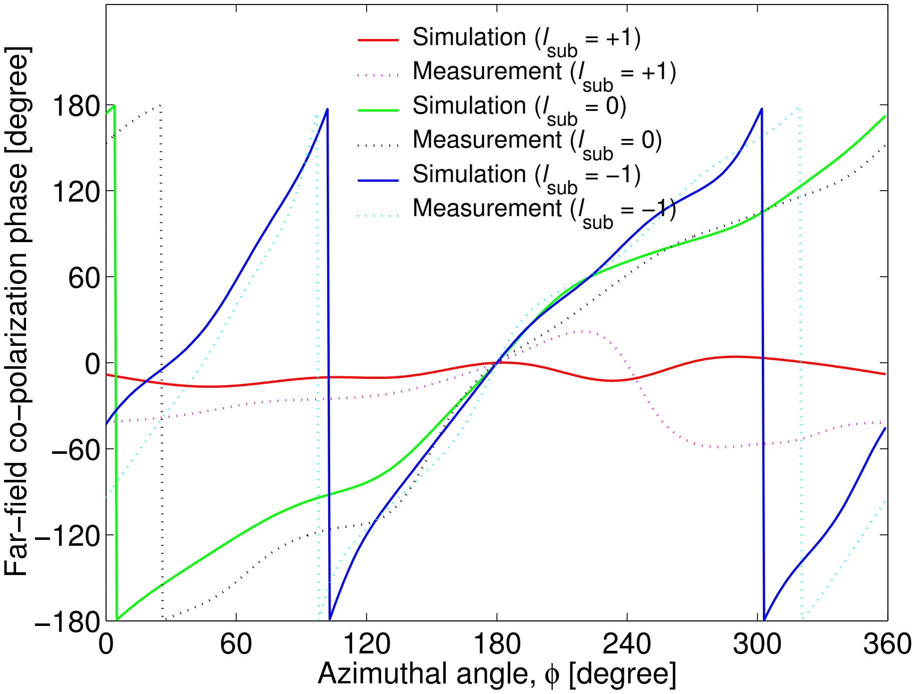

(b)

Fig. 7. Simulated and measured azimuthal radiation patterns of the Cassegrain dual-reflectarray antennas in Fig. 5 using the same parameters shown in Fig. 6. The radiation patterns for $l_{\text{sub}} = +1, 0, -1$ are simulated and measured at $\theta = 4^\circ, 5^\circ, 6^\circ$, respectively. The measured near-field to far-field transformation shown in Fig. 6 is again utilized for the comparison. A discrepancy between the simulation and measurement is mainly caused by the positional variation of the helical phase centers of $l_{\text{sub}} = \pm 1$. **(a)** Directivity. **(b)** Far-field co-polarization phase.

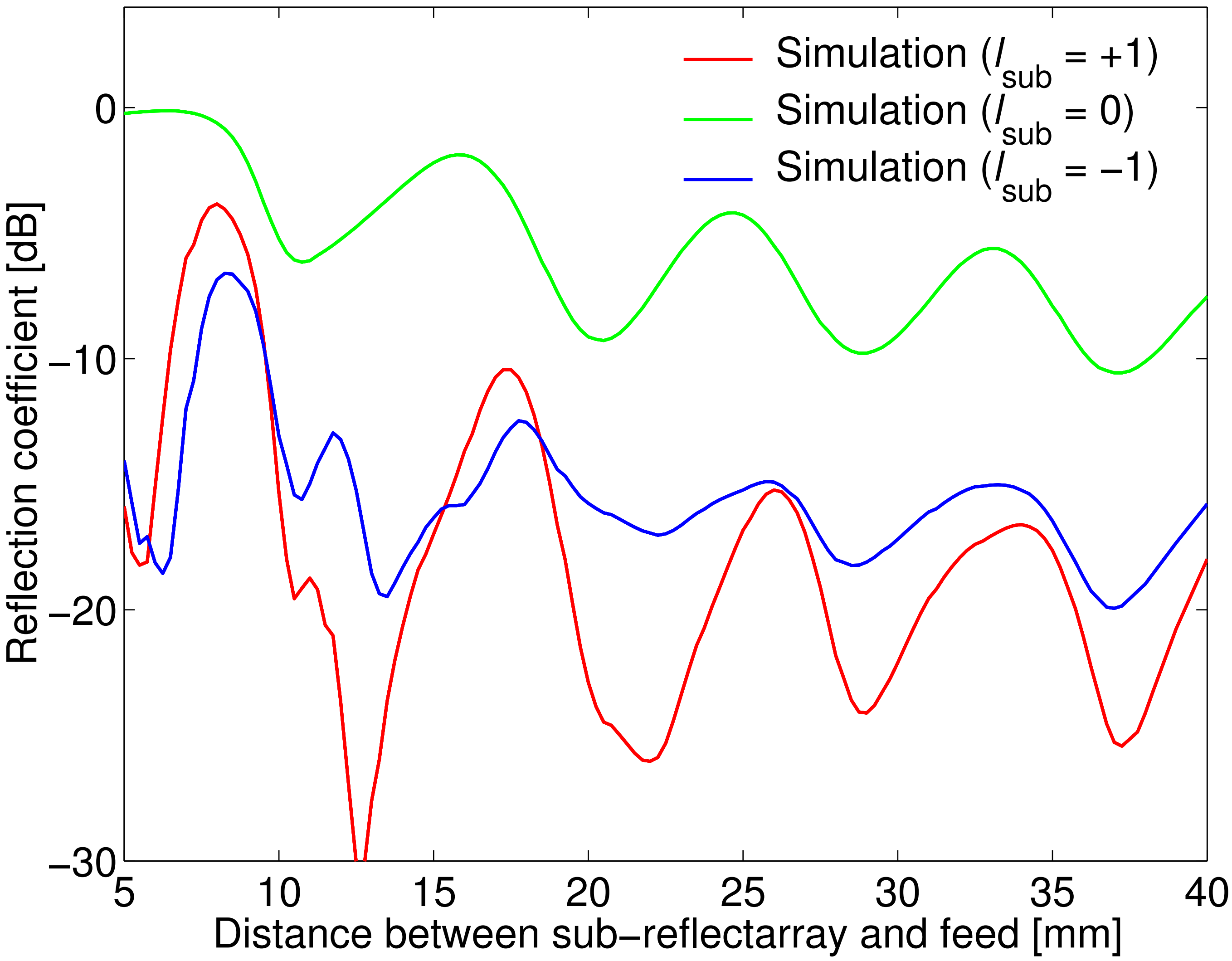

Fig. 8. Simulated reflection coefficients (S_{11}) versus distance between the sub-reflectarray and feed shown in Fig. 5 when $f = 18$ [GHz] and the main reflectarray in Fig. 5 is removed in the simulation setup. The S_{11} of $l_{\text{sub}} = \pm 1$ is always lower than that of $l_{\text{sub}} = 0$ owing to the OAM mode isolation.

III. Supplementary Measurement of a Cassegrain Dual-reflector Antenna

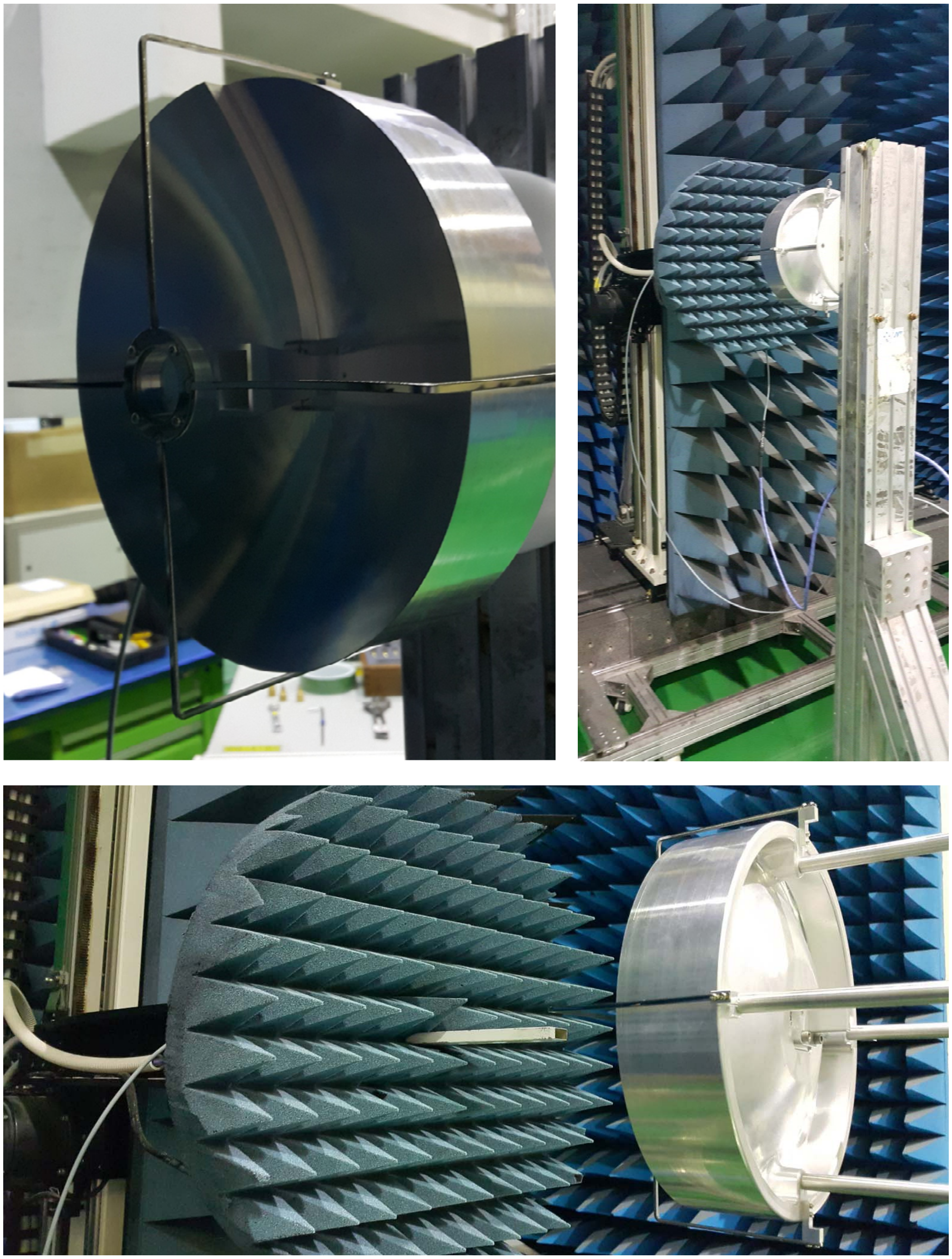

Fig. 9. Near-field antenna measurement setup for the fabricated Cassegrain dual-reflector antenna with helicoidal surfaces on the main ($l_{\text{main}} = -1$) and subreflectors ($l_{\text{sub}} = +1$). Based on [5] , the Cassegrain dual-reflector antenna was designed and fabricated to generate the total OAM mode number ($l_{\text{tot}} = l_{\text{main}} - l_{\text{sub}}$). Primary design parameters are as follows: f_m/D_m = 0.3, D_s/D_m = 0.2, -10dB beamwidth of the horn feed = 75°, and the diameter of the main reflector = 237 [mm], where f_m is the focus of the main reflector and D_m, D_s are the diameters of the main and subreflectors, respectively.

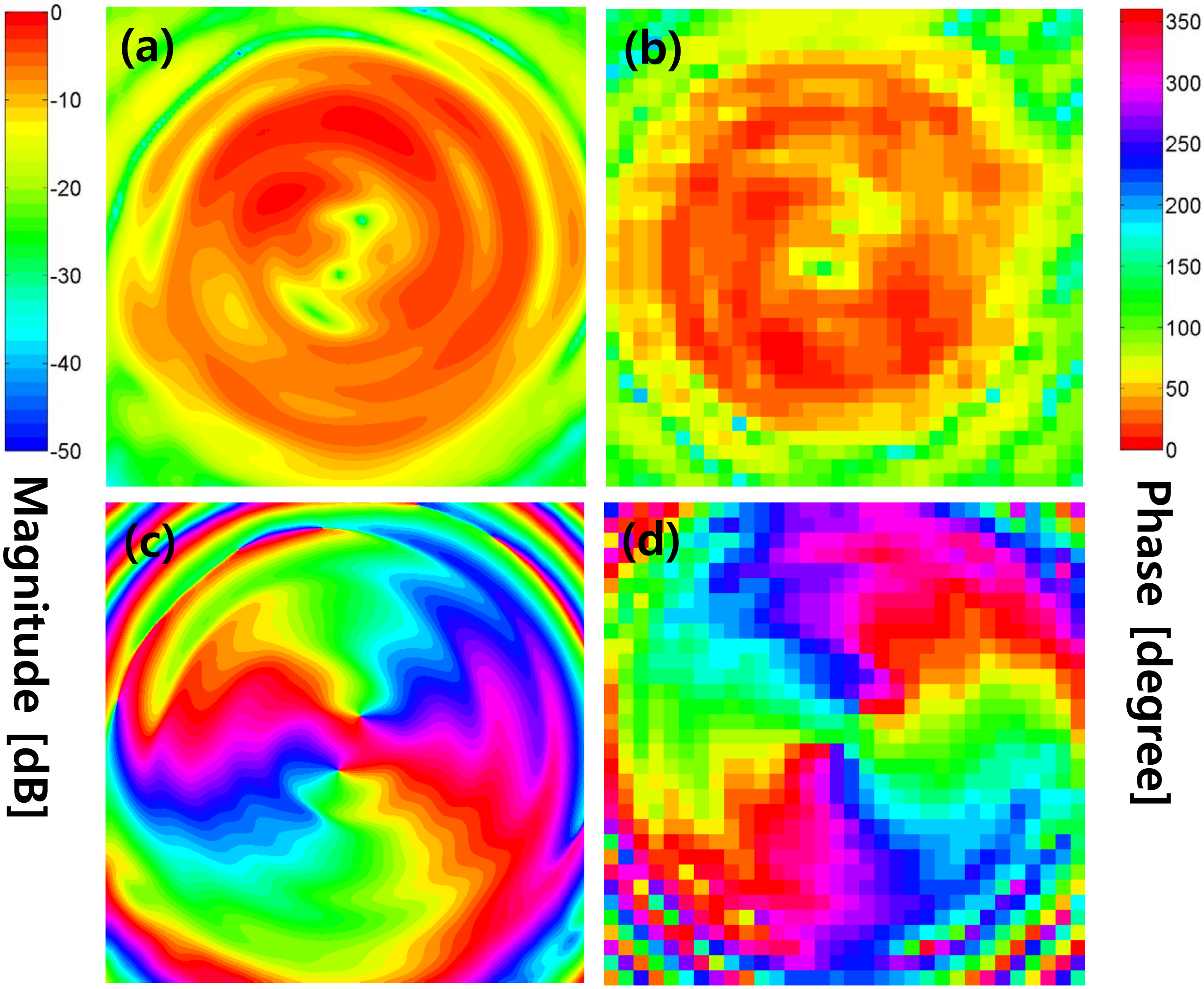

Fig. 10. Simulated and measured near-field magnitude and phase patterns of the Cassegrain dual-reflector antenna illustrated in Fig. 9 using $f = 18$ [GHz], $z = 107$ [mm], $l_{\text{main}} = -1$, and $l_{\text{sub}} = +1$. The edge of the main reflector is placed at $z = 0$. **(a)** Simulated magnitude pattern. **(b)** Measured magnitude pattern. **(c)** Simulated phase pattern. **(d)** Measured phase pattern.

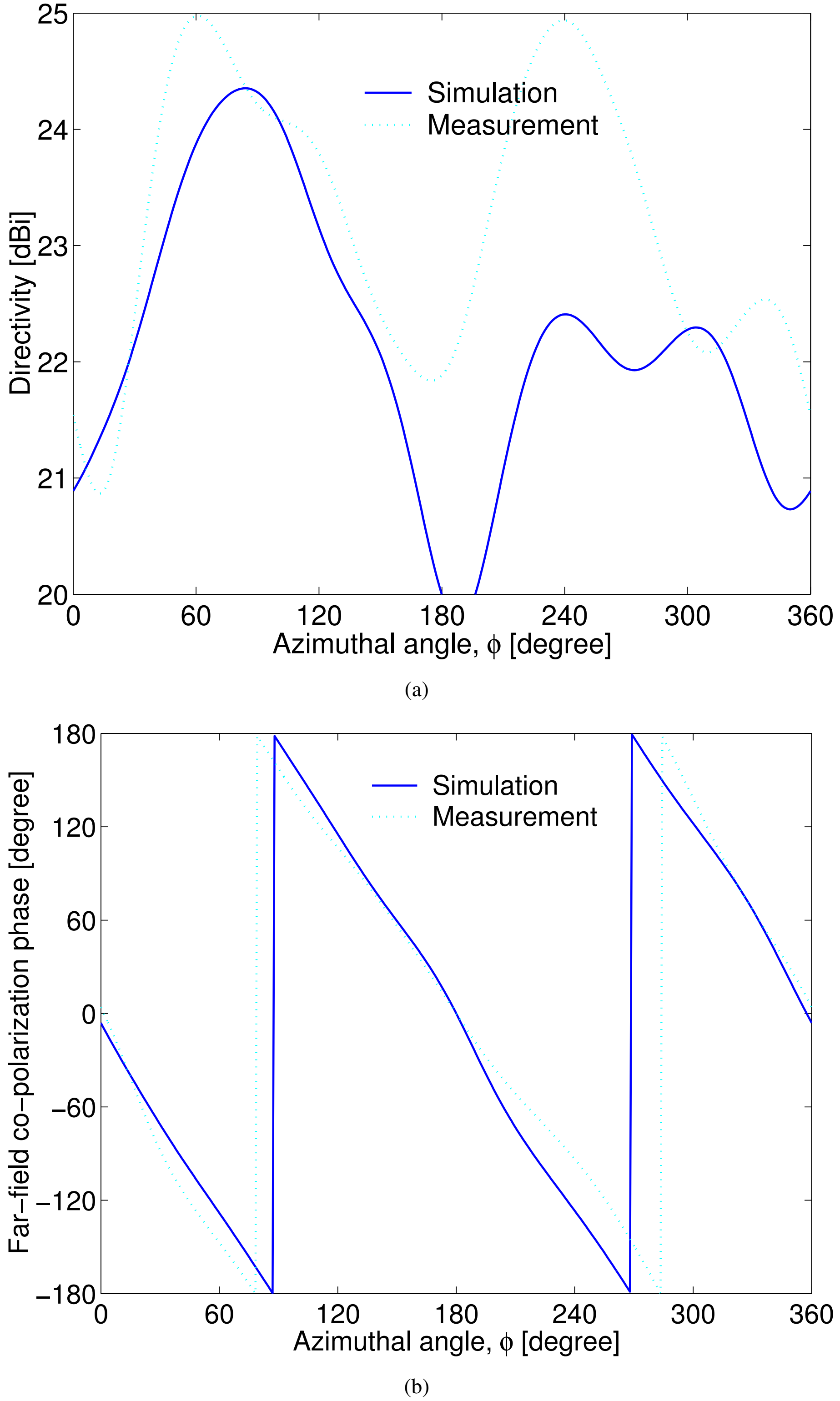

Fig. 11. Simulated and measured azimuthal radiation patterns of the Cassegrain dual-reflector antenna ($l_{\text{main}} = -1$ and $l_{\text{sub}} = +1$) in Fig. 9 using f = 18 [GHz] and $\theta = 6^{\circ}$. **(a)** Directivity. **(b)** Far-field co-polarization phase.

References

[1] D. G. Berry, R. G. Malech, and W. A. Kennedy, "The reflectarray antenna," *IEEE Trans. Antennas Propag.*, vol. 11, no. 6, pp. 645-651, Nov. 1963.

[2] J. Huang and J. A. Encinar, *Reflectarray Antennas*, Wiley-IEEE Press, 2007.

[3] C. A. Balanis, *Antenna Theory: Analysis and Design*, 3rd ed., Wiley-Interscience, 2005.

[4] B. Thidé, H. Then, J. Sjöholm, K. Palmer, J. Bergman, T. D. Carozzi, Ya. N. Istomin, N. H. Ibragimov, and R. Khamitova, "Utilization of photon orbital angular momentum in the low-frequency radio domain," *Phys. Rev. Lett.*, vol. 99, no. 8, pp. 087701, Aug. 2007.

[5] C. Granet, "Designing axially symmetric Cassegrain or Gregorian dual-reflector antennas from combinations of prescribed geometric parameters," *IEEE Antennas Propag. Mag.*, vol. 40, no. 2, pp. 76-82, April 1998.
